# Development of a Melting-Curve-Based Multiplex Real-Time PCR Assay for the Simultaneous Detection of Viruses Causing Respiratory Infection

**DOI:** 10.3390/microorganisms11112692

**Published:** 2023-11-02

**Authors:** Eliandro Reis Tavares, Thiago Ferreira de Lima, Guilherme Bartolomeu-Gonçalves, Isabela Madeira de Castro, Daniel Gaiotto de Lima, Paulo Henrique Guilherme Borges, Gerson Nakazato, Renata Katsuko Takayama Kobayashi, Emerson José Venancio, César Ricardo Teixeira Tarley, Elaine Regina Delicato de Almeida, Marsileni Pelisson, Eliana Carolina Vespero, Andrea Name Colado Simão, Márcia Regina Eches Perugini, Gilselena Kerbauy, Marco Aurélio Fornazieri, Maria Cristina Bronharo Tognim, Viviane Monteiro Góes, Tatiana de Arruda Campos Brasil de Souza, Danielle Bruna Leal Oliveira, Edison Luiz Durigon, Lígia Carla Faccin-Galhardi, Lucy Megumi Yamauchi, Sueli Fumie Yamada-Ogatta

**Affiliations:** 1 Laboratory of Molecular Biology of Microorganisms, Department of Microbiology, State University of Londrina, Londrina 86057-970, Brazil; tavares.eliandro@uel.br (E.R.T.); dgaiotto2@gmail.com (D.G.d.L.); 2 Graduate Program in Microbiology, Department of Microbiology, State University of Londrina, Londrina 86057-970, Brazil; ferreira.thiagodelima@gmail.com (T.F.d.L.); isabela.mcastro@uel.br (I.M.d.C.); pauloguilhermeph@gmail.com (P.H.G.B.); gnakazato@uel.br (G.N.); kobayashirkt@uel.br (R.K.T.K.); lgalhardi@uel.br (L.C.F.-G.); 3 Graduate Program in Clinical and Laboratory Pathophysiology, Department of Pathology, Clinical and Toxicological Analysis, State University of Londrina, Londrina 86038-350, Brazil; guilherme.bartolomeu@uel.br (G.B.-G.); emersonj@uel.br (E.J.V.); marsileni@uel.br (M.P.); elianacv@uel.br (E.C.V.); deianame@uel.br (A.N.C.S.); marciaperugini@uel.br (M.R.E.P.); 4 Graduate Program in Chemistry, Department of Chemistry, State University of Londrina, Londrina 86057-970, Brazil; tarley@uel.br; 5 Department of Pathology, Clinical and Toxicological Analysis, State University of Londrina, Londrina 86038-350, Brazil; elainedelicato@hotmail.com; 6 Graduate Program in Nursing, Department of Nursing, State University of Londrina, Londrina 86038-350, Brazil; gilselena@uel.br; 7 Graduate Program in Health Sciences, Department of Clinical Surgery, State University of Londrina, Londrina 86038-350, Brazil; marcofornazieri@gmail.com; 8 Department of Basic Health Sciences, State University of Maringá, Maringá 87020-900, Brazil; mcbtognim@uem.br; 9 Institute of Molecular Biology of Paraná, Curitiba 81350-010, Brazil; viviane@ibmp.org.br; 10 Carlos Chagas Institute, Oswaldo Cruz Foundation (FIOCRUZ-Pr), Curitiba 81350-010, Brazil; tatiana.brasil@fiocruz.br; 11 Albert Einstein Hospital, São Paulo 05652-900, Brazil; danielle.durigon@einstein.br; 12 Laboratory of Clinical and Molecular Virology, University of São Paulo, São Paulo 05508-000, Brazil; eldurigo@usp.br

**Keywords:** COVID-19, SARS-CoV-2, Influenza A virus, Human Respiratory Syncytial Virus, Human Rhinovirus B, nucleic acid amplification test

## Abstract

The prompt and accurate identification of the etiological agents of viral respiratory infections is a critical measure in mitigating outbreaks. In this study, we developed and clinically evaluated a novel melting-curve-based multiplex real-time PCR (M-m-qPCR) assay targeting the RNA-dependent RNA polymerase (RdRp) and nucleocapsid phosphoprotein N of SARS-CoV-2, the Matrix protein 2 of the Influenza A virus, the RdRp domain of the L protein from the Human Respiratory Syncytial Virus, and the polyprotein from Rhinovirus B genes. The analytical performance of the M-m-qPCR underwent assessment using in silico analysis and a panel of reference and clinical strains, encompassing viral, bacterial, and fungal pathogens, exhibiting 100% specificity. Moreover, the assay showed a detection limit of 10 copies per reaction for all targeted pathogens using the positive controls. To validate its applicability, the assay was further tested in simulated nasal fluid spiked with the viruses mentioned above, followed by validation on nasopharyngeal swabs collected from 811 individuals. Among them, 13.4% (109/811) tested positive for SARS-CoV-2, and 1.1% (9/811) tested positive for Influenza A. Notably, these results showed 100% concordance with those obtained using a commercial kit. Therefore, the M-m-qPCR exhibits great potential for the routine screening of these respiratory viral pathogens.

## 1. Introduction

Historically, mankind has witnessed the emergence of infectious agents, some of which have unleashed devastating effects on the human population, such as those responsible for the smallpox and influenza pandemics [1]. Given their ease of transmission via aerosol, respiratory viral pathogens have been implicated as potential catalysts of pandemics, as exemplified in the past two decades by the emergence of severe acute respiratory syndrome coronavirus (SARS-CoV) in 2002; H1N1 Influenza A in 2009; Middle East respiratory syndrome coronavirus (MERS-CoV) in 2012; and most recently, SARS-CoV-2 in 2020 [2,3]. In addition to the route of transmission, respiratory viruses share several common characteristics: (i) their capacity to affect healthy and immunocompromised individuals of all ages; (ii) the substantial global burden of epidemics caused by these viruses, amounting to millions of cases annually; (iii) the propensity of viral RNA polymerases to exhibit a low fidelity rate, leading to point mutations in each replication cycles [4] and the subsequent emergence of variants; (iv) the ability to cause infections that can manifest as either symptomatic or asymptomatic cases; (v) their potential to induce elevated rates of hospitalization, morbidity, and mortality, especially among young children, the elderly, and adults with underlying chronic diseases; (vi) the significant economic impact they impose, encompassing treatment costs, absenteeism from work/school, and productivity losses [5,6,7,8,9].

SARS-CoV-2, an enveloped single-stranded positive-sense RNA virus belonging to the *Coronaviridae* family [3], is the causative agent of COVID-19 (Coronavirus Disease-19), a potentially fatal severe acute respiratory syndrome characterized by high rates of transmission and infection. Since its initial identification in 2019 in Wuhan, this disease has affected more than 771 million individuals worldwide, with cumulative deaths surpassing 6.9 million [2]. Both symptomatic and asymptomatic patients exhibit a high viral load in the nostrils shortly after infection onset, highlighting their crucial role in the virus transmission chain and the importance of detecting SARS-CoV-2 within the population [10,11]. The success in containing the spread of COVID-19 has been attributed to a set of non-pharmacological measures such as social distancing, mask-wearing, rigorous hand hygiene, travel restrictions, and temporary school closures. Furthermore, extensive diagnostic testing to detect SARS-CoV-2 within the population and subsequent vaccination campaigns have played pivotal roles in managing the pandemic. However, along with the intrinsic characteristics of viral RNA polymerases, the viral genome can undergo recombination and reassortment, contributing to high diversity and the emergence of new variants. Thus, the availability of assays for diagnosing/monitoring the infectious agent remains an essential component of infection control strategies [12].

Generally, the most common symptoms of COVID-19 are indistinguishable from those associated with other viral agents causing respiratory infections. These symptoms include fever, headache, cough, muscle ache, fatigue, dyspnea, and loss of smell and taste [3,13]. Therefore, syndromic diagnosis cannot identify specific viral pathogens responsible for respiratory tract infections. In fact, epidemics of viral respiratory tract infections caused by non-coronaviruses remain highly prevalent worldwide and can lead to severe consequences in susceptible individuals. Notable examples of such viral agents include the influenza (FLU) virus, Human Respiratory Syncytial Virus (HRSV), and Human Rhinovirus B (HRV-B).

Epidemiological studies have shown that most viruses responsible for respiratory tract infections exhibit seasonal patterns. Specifically, FLU and HRSV tend to be more prevalent during winter, while HRV-B can be detected throughout the year [8]. Nevertheless, the COVID-19 pandemic has impacted the epidemiology of viral infections, including the genetic diversity of these non-SARS-CoV-2 viral agents during the pandemic. A significant decrease in respiratory infections and hospitalizations caused by non-SARS-CoV-2 viruses has been observed globally [14,15,16]. Despite this decrease, cases of co-infection involving SARS-CoV-2 and these viral agents have also been reported during the COVID-19 pandemic [17,18,19].

Seasonal influenza is primarily caused by the influenza virus, mainly of type A (FLU-A), which circulates worldwide. This enveloped virus consists of a single strand of RNA divided into eight negative-sense RNA segments and belongs to the *Orthomyxoviridae* family. The severity of the disease ranges from mild to severe, with specific populations at a higher risk of developing severe illness. These include pregnant women, younger children, the elderly, and individuals with underlying chronic conditions such as pulmonary, cardiac, renal, metabolic, liver, or hematologic diseases. Additionally, individuals with immunosuppressive conditions, including those undergoing chemotherapy or steroid treatment or with malignancies, are also more susceptible to severe outcomes [6]. According to the World Health Organization (WHO), the annual epidemic burden of severe influenza illnesses is estimated to be around 3 to 5 million cases, resulting in approximately 290,000 to 650,000 deaths [20]. Control measures for FLU-A infections include vaccines and antiviral drugs [21]. However, akin to SARS-CoV-2, new subtypes and variants of FLU-A emerge frequently, and occasionally, a new human strain may arise from an animal source, thereby posing a potential threat to human populations [6,22].

Human Respiratory Syncytial Virus (HRSV), an enveloped single-strand, negative-sense RNA virus belonging to the *Pneumoviridae* family, can cause the common cold in individuals of all ages. However, it is particularly notorious as a leading cause of lower respiratory tract infection in young children [5,23]. In this vulnerable population, HRSV infection may progress to potentially fatal conditions such as bronchiolitis and community-acquired pneumonia [5]. Furthermore, adults with underlying diseases (including chronic pulmonary or circulatory diseases and functional disability) and the elderly also face a heightened risk of experiencing severe HRSV infections [24]. Currently, only two HRSV vaccines have been approved by the U.S. Food and Drug Administration for use in adults over 60 years [5,25]. Moreover, the first monoclonal antibody was recently approved to prevent HRSV infections in children [5].

Rhinovirus (HRV), a non-enveloped single-strand, positive-sense RNA virus of the *Picornaviridae* family, has been identified as the primary causative agent of the common cold, particularly affecting children [7,26,27]. Typically, infections caused by this virus exhibit a mild and self-limiting course; however, they can also progress to severe manifestations, including community-acquired pneumonia [26], with a high risk of death [5]. Moreover, HRV is important in asthma exacerbation [28]. Unfortunately, despite medical advancements, no approved vaccines or other preventive therapies are available for HRV infection [5].

The early and accurate identification of viral pathogens causing respiratory infections is crucial to control their spread and provide specific and immediate treatment, thus avoiding outbreaks and complications for patients [29]. While isolation and identification of the infectious agent are considered standards for clinical laboratory diagnosis, it is essential to acknowledge that these techniques are labor-intensive, time-consuming, and possess limited sensitivity [30]. Conversely, nucleic acid amplification tests (NAATs) have demonstrated remarkable specificity and sensitivity in detecting the etiological agents of infections, surpassing conventional culture-based testing methods [12,31]. Multiplex polymerase chain reaction (PCR)-based tests, for instance, enable the simultaneous detection of several molecular markers using distinct pairs of oligonucleotide primers. Amplicons can be detected either through agarose gel electrophoresis or in real-time (m-qPCR) using DNA-intercalating fluorophores during PCR or specific probes complementary to the target gene.

Real-time PCR (qPCR) remains the gold standard for the detection of SARS-CoV-2 and other respiratory viruses in clinical and environmental samples, and most in-house and commercial tests rely on the use of fluorescent probes [31,32,33,34,35]. This study aimed to develop a melting-curve-based multiplex real-time PCR assay (M-m-qPCR) for the simultaneous detection of four respiratory viral pathogens: SARS-CoV-2, FLU-A, HRSV, and HRV-B. This assay targets specific genes, including the RNA-dependent RNA polymerase (RdRp) and the nucleocapsid phosphoprotein N of SARS-CoV-2, the Matrix protein 2 of FLU-A, the RdRp domain of the L protein from HRSV, and the polyprotein from HRV-B. The potential usefulness of this assay was evaluated using nasopharyngeal swab specimens.

## 2. Materials and Methods

### 2.1. Oligonucleotide Primers and Positive Controls

The nucleotide sequences of the genes encoding RdRp (gene: ORF1ab) and nucleocapsid phosphoprotein N (gene: N) of SARS-CoV-2, Matrix protein 2 (gene: M2) of FLU-A, the RdRp domain of the L protein (gene: L) of HRSV, and the polyprotein (gene: 5′ untranslated region-UTR) of HRV-B were obtained from the GenBank/EMBL databases (available on the website http://www.ncbi.nlm.nih.gov accessed on 24 August 2020). These sequences were analyzed using the BioEdit v.7.2.0 software. Specific primers were designed based on the consensus sequence of each gene, employing the OligoAnalyzer^™^ tool (http://www.idtdna.com, accessed on 28 August 2020). Additionally, primers targeting the human tRNA-processing ribonuclease P (RNase P) gene [34,36] were also included in this study. Detailed information on the primer sequences and the expected size of the amplicons can be found in Table 1. To create positive controls, the consensus sequences of each viral target were inserted into plasmid pUC57 (Figure S1, FastBio Ltd.a, Ribeirão Preto, Brazil).

### 2.2. Viral and Microbial Strains

A panel comprising 47 viral strains, 12 bacteria, and 10 fungi (Table 2) was used to develop the assays. This panel encompassed various microbial and viral species components of the respiratory tract microbiota or pathogens associated with this anatomical site. Three colony-forming units (CFUs) of each bacterial and fungal species were cultivated at 37 °C for 24 h in tryptic soy broth (TSB, Oxoid, São Paulo, Brazil) and Sabouraud dextrose broth (SDB, Himedia, Thane, India), respectively. Following cultivation, the microbial cells were harvested via centrifugation (10,000× *g* for 5 min), washed twice with sterile PBS, and processed for DNA purification. Bacterial and fungal cultures were kept at −80 °C in TSB containing 20% glycerol and SDB containing 20% glycerol, respectively. Viruses were obtained from the viral collection of the Laboratory of Virology (LAVIR) of the State University of Londrina (UEL) and the Laboratory of Clinical and Molecular Virology of the University of São Paulo.

### 2.3. Nucleic Acid Purification

The QIAamp^®^ DNA Mini kit (QIAGEN, São Paulo, Brazil) and QIAamp^®^ Viral RNA Mini kit (QIAGEN, São Paulo, Brazil) were used for DNA and RNA purification, respectively, according to manufacturer’s recommendations.

### 2.4. PCR Design

The amplification reaction conditions were determined through a two-step process. First, the annealing temperatures and primer concentrations were established via conventional PCR. Subsequently, the established conditions were tested in qPCR assays. Therefore, each primer pair, at concentrations ranging from 0.5 to 2 μM, was used in conventional PCRs with a final volume of 20 µL. The reaction mix contained 20 mM of Tris-HCl (pH 8.4), 5 mM of KCl, 1.5 mM of MgCl_2_, 100 μM of each dNTP, 2.5 U of *Taq* DNA polymerase (Invitrogen, São Paulo, Brazil), and 1 × 10^6^ copies of the positive control. The amplification reactions were performed in a Veriti 96-well Thermal Cycler (Applied Biosystems, São Paulo, Brazil) with an initial denaturation at 95 °C for 1 min, followed by 35 cycles of 95 °C for 30 s, an annealing temperature gradient ranging from 60 °C to 70 °C for 1 min, and an extension step at 72 °C for 45 s. Negative template control (NTC) reactions without template nucleic acid were carried out simultaneously. Subsequently, amplicons were analyzed via 3% agarose gel electrophoresis after staining with GelRed^®^ (Biotium-Uniscience, Osasco, Brazil).

An annealing temperature of 61 °C and a primer concentration of 1 μM were selected to validate the optimized conditions in the qPCR assays, using the positive controls and nucleic acid purified of each viral strain as templates. Thus, all qPCRs were performed using a Rotor-Gene Q 5PlexHRM (QIAGEN, Hilden, Germany) in a final volume of 20 µL, which contained 1 µM of each viral primer pair, 1 µM of human RNase P primers, and QuantiNova SYBR^®^ Green RT-PCR mix (QIAGEN, São Paulo, Brazil), following the manufacturer’s recommendations. The cycling conditions were as follows: an initial denaturation at 95 °C for 2 min, followed by 40 cycles of 95 °C for 30 s, 61 °C for 30 s, and 72 °C for 30 s. Melting curves were acquired using 0.5 °C steps with a hold of 60 s at each step, ranging from 60 to 99 °C. NTC reactions were carried out simultaneously. Data were analyzed using the Rotor-Gene Q series software version 2.1.0.9.

### 2.5. Analytical Specificity, Sensitivity, and Performance in Virus-Spiked Swabs

The specificity of the M-m-qPCR was assessed using 100 ηg of nucleic acid obtained from cultures or clinical samples of a panel of bacteria, fungi, and viruses (Table 2). All amplification reactions were performed in duplicate in three independent experiments. Moreover, the primer sequences targeting the selected genes were compared with nucleotide sequences available in the GenBank databases of the National Center for Biotechnology Information (NCBI, http://www.ncbi.nlm.nih.gov accessed on 28 August 2020) using the Blast algorithm (blastn).

The sensitivity of the M-m-qPCR was empirically determined using positive controls ranging from 10^0^ to 10^6^ copies per reaction. Each strain was processed in triplicate on five consecutive days. For each primer pair, a standard curve was generated from the C_T_ values as a function of the positive plasmid copy number, and the R^2^ was calculated to evaluate the efficiency of the reaction. The slope of this line was used to determine the efficiency (E) according to the following equation: E = 10^−1/slope^ − 1. The sensitivity of the M-m-qPCR was also determined using SARS-CoV-2 RNA. Therefore, Vero ATCC CCL81 (Merck, São Paulo, Brazil) cells were cultured in Dulbecco’s modified Eagle’s medium (DMEM, Invitrogen-Gibco, Waltham, MA, USA), supplemented with 10% fetal bovine serum (Invitrogen-Gibco), glutamine (2 mM, Sigma-Aldrich, St. Louis, MI, USA), streptomycin (100 µg/mL, Gibco BRL, Waltham, MA, USA), and penicillin (100 IU/mL, Novafarma Ind. Farm., Anápolis, Brazil). SARS-CoV-2/human/BRA/SP02cc/2020 stock was obtained via inoculation in Vero cells until a cytopathic effect was achieved (CPE ~90%) and stored at −80 °C. The virus titration was performed using a median tissue culture infectious dose (TCID_50_/mL) assay [37]. Cells were seeded into 96-well plates (5 × 10^4^ cells/mL) 24 h before the experiment. Viruses were 10-fold serially diluted in DMEM (10^−1^ to 10^−12^). The medium was removed from the cell culture plates, and then, virus dilutions were added in sextuplicate and incubated at 37 °C. Visualizations were performed daily in an inverted light microscope (Axiovert 100, Carl Zeiss, Oberkochen, Germany) to observe the CPE over 72 h. The viral titer was calculated using the Spearman and Kärber algorithm [38] and expressed in TCID_50_/mL. The viral titer was determined as 1.44 × 10^7^ TCID_50_/mL. The limit of detection (LoD) was established using the SARS-CoV-2 viral concentration (Genomic Copy Equivalents—GCEs) ranging from 5 to 10,000 GCEs.

The performance of the M-m-qPCR was analyzed using simulated swabs spiked with the viruses. Initially, swabs were soaked in a 2% polyethylene oxide (*w/v*) solution that mimics mucus [39]. Afterward, 100 µL aliquots of each supernatant from virus-infected cells with cytopathic effects were used to spike the swabs, which were further processed for RNA extraction as described above.

### 2.6. Performance of the Multiplex Real-Time PCR Assay in Clinical Samples

The performance of the M-m-qPCR was initially evaluated using nasopharyngeal samples collected from 20 patients who sought care for respiratory syndromes at reference centers in Londrina, Paraná, Brazil, and tested positive for COVID-19. Nasopharyngeal secretions were collected using Rayon swabs (Inlab, São Paulo, Brazil) and maintained in viral transport medium (DMEM containing 1000 IU/mL penicillin, 1000 μg/mL streptomycin, and 25 μg/mL amphotericin B). Patients were randomly selected according to a C_T_ value < 21 detected using the TaqPath^™^ COVID-19 CE-IVD RT-PCR kit and the 7500 Real-Time PCR System (ThermoFisher Scientific, Waltham, MA, USA) for SARS-CoV-2 detection. The viral species were further confirmed through genome or Spike protein-encoding gene (gene S) sequencing. The sequencing library preparation for the Illumina platform was performed using the Nextera XT Kit (Illumina, San Diego, CA, USA), according to the manufacturer’s instructions. Libraries were quantified using the Qubit fluorimetric method (ThermoFisher Scientific, Waltham, MA, USA) and sequenced on the NextSeq 550 instrument with 300 paired-end cycle kits (Illumina, San Diego, CA, USA) at the Hospital Israelita Albert Einstein, São Paulo, Brazil. The data underwent filtering and trimming to achieve a Phred score of <20. The genome was assembled using an “ab initio” strategy with reference genome NC_045512.2 (SAR-CoV-2) and the SPADES software, v.3.13.1. Whole-genome consensus sequences were classified using the Phylogenetic Assignment of Named Global Outbreak Lineages (PANGOLIN) software, v3.1.18 (pangolearn 2022-01-20, constellations v0.1.2, scorpio v0.3.16, and pango-designation release v1.2.123) [40]. The whole-genome sequences of the viral strains were submitted to DDBJ/ENA/GenBank under the submission number SUB12140327.

Next, the performance of the M-m-qPCR for SARS-CoV-2 in clinical samples was compared with the results obtained from the Molecular SARS-CoV-2 (E)—Bio-Manguinhos kit (FIOCRUZ, Curitiba, Brazil). This comparison involved 811 individuals who exhibited symptoms of respiratory infection and/or had contact with people diagnosed with COVID-19 between November 2020 and May 2023. The study protocol was approved by the Ethics Committee of the UEL (Document 47784621.2.0000.5231, Opinion Number 4.862.243—CEP/UEL). Written informed consent was obtained from all participants, including their agreement with the publication of this report and any accompanying images. Nasopharyngeal swabs from each participant were collected using Rayon swabs (Inlab, São Paulo, Brazil). The swabs were transferred to a tube containing 560 µL of lysis buffer from the QIAamp^®^ Viral RNA Mini kit (QIAGEN, São Paulo, Brazil) and incubated at room temperature for 30 min before undergoing nucleic acid purification, as recommended by the manufacturer.

## 3. Results and Discussion

### 3.1. Melting-Curve-Based Multiplex Real-Time PCR Assay

NAATs have been used in many clinical laboratories to diagnose different infectious diseases because of their high specificity and sensitivity, as well as their ability to provide rapid results. However, culture-based tests to identify viral agents, along with many microorganisms, are known for their time-consuming and labor-intensive nature, in addition to their limited sensitivity [30]. Moreover, several clinically relevant viruses are challenging to cultivate in vitro [30] or require a high biosafety level (BSL) laboratory for handling, such as SARS-CoV-2 (requiring BSL-3 for viral culture versus BSL-2 for NAATs) [34].

**Figure 1:**
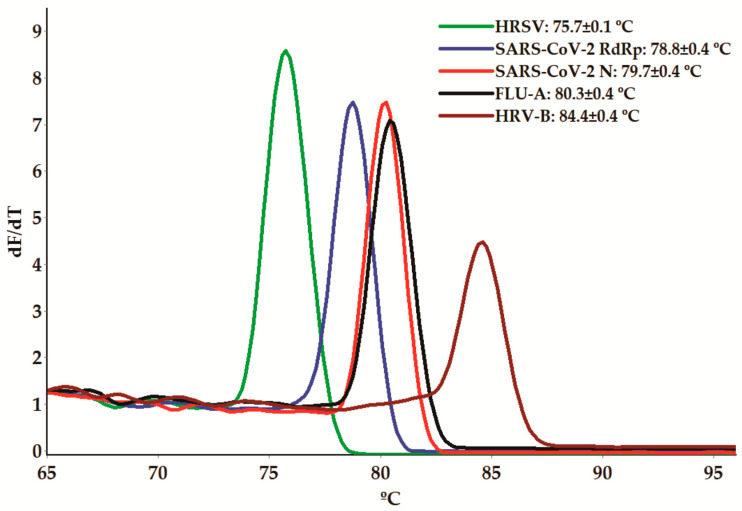
Melting curve analyses showing the melting temperature (Tm) peaks of RNA-dependent RNA polymerase (RdRp) and the nucleocapsid phosphoprotein N of SARS-CoV-2, the Matrix protein 2 of the Influenza A virus (FLU-A), the RdRp domain of the L protein from Human Respiratory Syncytial Virus (HRSV), and the 5′-UTR polyprotein from Human Rhinovirus B (HRV-B) amplicons using the positive (plasmid) controls.

The main outcome of our study is methodological. We developed a melting-curve-based real-time multiplex-PCR assay using SYBR green dye, allowing for the simultaneous detection of SARS-CoV-2, FLU-A, HRSV, and HRV-B (Figure 1). To enhance the specificity and accuracy of the assays, our selection of target nucleotide sequences was based on the following criteria: (i) essential gene status; (ii) conserved sequences within the species to avoid false-negative results due to genetic variations; (iii) non-overlapping melting temperature (Tm) ranges for the different amplicons within the assay; (iv) the presence of secondary structures and dimerization (self- and heterodimerization) not exceeding 10% of the Tm and total ∆G values, respectively, of the primers.

These genes have been studied as potential therapeutic targets for the development of antiviral agents [4,41,42,43,44], reinforcing their crucial role in viral life cycles. For SARS-CoV-2, genes encoding RdRp (ORF1ab) and N (gene: N) proteins were selected for primer design in this study. These genes have been recognized as reliable targets for NAATs [45,46,47]. Indeed, most available NAATs for detecting SARS-CoV-2 contain redundancies to avoid targeting failures resulting from viral genomic mutations [45]. Notably, a high degree of structural conservation has been observed among viral RdRps [48]. Nonetheless, variations in RNA binding, polymerization, and the presence of accessory domains have also been noted among these enzymes [4]. The N gene, conversely, is highly conserved, sharing over 98% similarity across various SARS-CoV-2 variants, including Wuhan (China), Alpha (UK), Beta (South Africa), Gamma (Brazil), Delta (India), Epsilon (USA), and Omicron (South Africa) [43]. The N protein is the most abundant in virions and plays several essential roles in the SARS-CoV-2 life cycle, including transcription, the replication of the viral genome, the encapsidation of the viral genome into a ribonucleotide complex, and assembly into viral particles [47].

The L protein of HRSV contains an RdRp domain (RNA transcription/replication), a polyribonucleotidyltransferase (cap addition) domain, and a methyltransferase (cap methylation) domain, all of which are indispensable for the viral replicative cycle. Notably, the C-terminal region of the L protein is the most variable domain among non-segmented negative-strand viruses [44].

Regarding HRV-B, its genome consists of a positive-sense single-strand RNA of approximately 7200 bp that encodes a single open reading frame (ORF). The 5′-terminal sequence (80 to 84 bases) exhibits minimal structural variation across species and displays a cloverleaf-like (CL) motif, which interacts with viral and cellular proteins to initiate RNA synthesis. Immediately following the CL motif, all Rhinoviruses share unique sequences consisting of a pyrimidine-rich spacer segment with short oligo C (Cytosine) and oligo U (Uracil) units interspersed with Adenines [49]. In our study, the specific primers for HRV-B detection target the 5′-UTR polyprotein, a region commonly employed for the detection of this virus in clinical samples [7].

Finally, the M2 protein of FLU-A, an integral membrane protein that forms a pH-regulated ion channel, plays an essential role in viral replication and can contribute to host pathogenicity by interfering with cellular homeostasis or interacting with and modulating the host proteome [42].

To establish the amplification conditions for detecting these genes, we initially conducted conventional monoplex PCRs using synthetic viral controls (positive plasmid controls). Each specific primer pair (1 µM) successfully generated amplicons of the expected sizes, as shown in Table 1, with an annealing temperature of 61 °C. The identity of each amplicon was further confirmed via sequencing and searching for nucleotide sequence homology in the GenBank/EMBL databases.

Afterward, we used the positive controls to determine the equivalent Tms of each primer in the monoplex qPCR assays. All primer pairs successfully amplified the corresponding genes, generating dissociation curves with a single peak (Figure S2). The Tm values of all amplicons are presented in Figure 1 and Figure S2. These Tm values were further confirmed in monoplex qPCR using RNA purified from viral cultures. To evaluate the quality of the nucleic acid purification and the presence of potential PCR-interfering substances, primers targeting the human tRNA processing ribonuclease P (RNase P) gene were also included [34,50], resulting in a Tm value of 82.1 ± 0.50 °C (Figure S2).

### 3.2. Analytical Performance of the Assay

The specificity of each primer pair targeting the selected genes was initially analyzed in silico using the GenBank/EMBL database on the NCBI homepage. No matches were found other than those with the corresponding genes of specific viral species and the human genome, indicating that amplification signal of non-target sequences that result in cross-reactivity is not likely to occur. Subsequently, the specificity of the M-m-qPCR was confirmed using nucleic acid from the panel of bacteria, fungi, and viruses (Table 2). Amplification signals were detected only for the specific viral agents, demonstrating no cross-reactivity among non-target species.

**Figure 2:**
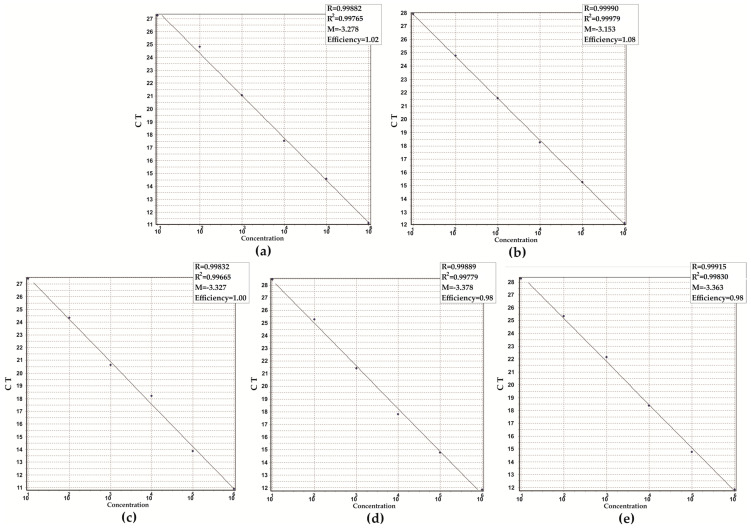
Sensitivity of multiplex real-time PCR assays. (**a**) RNA-dependent RNA polymerase (ORF1a/b) of SARS-CoV-2; (**b**) nucleocapsid phosphoprotein N (gene N) of SARS-CoV-2; (**c**) Matrix protein 2 (gene M2) of Influenza A virus; (**d**) RdRp domain of L protein (gene L) from Human Respiratory Syncytial Virus; (**e**) polyprotein (5′-UTR-polyprotein) from Human Rhinovirus B. Amplification plot of tenfold serial dilution corresponding to 10^1^ to 10^6^ copies of the positive controls. Standard curve represented by linear regression line for threshold cycle (C_T_) versus copy numbers. Slope (M), regression coefficient (R), and efficiency of the real-time PCR method are shown (**a**–**e**).

To assess the linearity and limit of detection (LoD) of the M-m-qPCR for the target genes, tenfold serial dilutions ranging from 10^0^ to 10^6^ copies per reaction of each positive control from specific viral species were prepared. Each concentration was analyzed in six replicates every day for five days (*n* = 30). The LoD of the m-qPCR for the target genes was 10 copies per reaction, and the reaction efficiencies, calculated from the slope of the standard curves, ranged from 98 to 100% (Figure 2). The mean C_T_ values of the target genes, using the LoD, were 29.8 (Orf1a/b) and 28.7 (N) for SARS-CoV-2, 29.7 (5′-UTR-polyprotein) for HRV-B, 28.1 (L) for HRSV, and 27.3 (M2) for FLU-A.

The sensitivity of M-m-qPCR was also evaluated using SARS-CoV-2 (SARS-CoV-2/human/BRA/SP02cc/2020 strain) RNA. The LoD of the assay for the RdRp- and N-encoding genes of SARS-CoV-2 was 5 GCEs, displaying C_T_ values of 22.6 (RdRp) and 23.6 (N) (Figure S3). These slight differences can be explained by the viral titration method used. The median TCID_50_ is defined as the dilution of a virus required to infect 50% of a given cell culture and is widely used for determining viral infectivity [51]. Therefore, it is possible that a higher load of viral particles was assessed in this assay.

According to these results, the M-m-qPCR is considered positive for all viruses when amplification signals for each specific gene target, as well as the control, are detected with C_T_ values ≤ 30 using the Rotor-Gene Q 5PlexHRM System. The performance of the M-m-qPCR is comparable to that exhibited by the two commercial kits that were used in this study. The TaqPath™ COVID-19 CE-IVD RT-PCR kit targets the genes encoding the ORF1ab, N protein, and S protein of SARS-CoV-2. According to the technical note, the LoD of the assay is 10 GCEs, with C_T_ cut-off values of ≤ 37 for all viral targets. The valid C_T_ values for the internal control (MS2 phage) are ≤ 32 using the 7500 Real-Time PCR System. On the other hand, the Molecular SARS-CoV-2 (E)—Bio-Manguinhos kit targets the genes encoding the ORF1ab and the envelope protein (E), and the LoD of the assay is 304 GCEs. The test is considered positive for SARS-CoV-2 when amplification signals for E and RdRp targets, as well as the internal control (RNase P), are detected with C_T_ values of ≤40, ≤35, or ≤37, respectively, also using the 7500 Real-Time PCR System. In addition to the primers, both kits use fluorescent probes as an amplification detection system.

Next, the performance of the M-m-qPCR assay was analyzed using a swab saturated with synthetic nasal mucus and spiked with the target viruses. Amplification signals were detected for all viruses, and the dissociation curves generated melting peaks corresponding to each specific target, as generated for the positive controls.

Based on these data, the optimized M-m-qPCR assay consists of a reaction mixture with a final volume of 20 µL. Tube 1 contains 1 µM of forward and reverse primers targeting the genes encoding the SARS-CoV-2 RdRp and N proteins, 2 × 10^3^ copies of the positive control, and a QuantiNova SYBR^®^ Green RT-PCR mix. Tube 2 includes 1 µM of forward and reverse primers targeting the HRV-B 5′-UTR-polyprotein, the HRSV RdRp domain of the L protein, the FLU-A Matrix protein 2 encoded genes, 2 × 10^3^ copies of the positive control, and a QuantiNova SYBR^®^ Green RT-PCR mix. In Tube 3, the reagents include 1 µM of forward and reverse primers targeting the human RNase P-encoded gene and a QuantiNova SYBR^®^ Green RT-PCR mix. In all tubes, 5 µL of template nucleic acid is added, and the final volume is adjusted with deionized water. NTC reactions, which lack any template nucleic acid, are carried out simultaneously.

### 3.3. Performance of the Assay in Clinical Samples

To assess the performance of the M-m-qPCR for SARS-CoV-2 detection in clinical samples, genomic RNAs extracted from nasopharyngeal specimens collected from 20 COVID-19-positive patients (C_T_ < 21) diagnosed with the TaqPath^™^ COVID-19 CE-IVD RT-PCR kit were tested with the M-m-qPCR. The developed assay was capable of detecting all SARS-CoV-2 strains (Table 3). In addition, viral genome sequencing conducted as part of the study showed that the standardized M-m-qPCR assay could detect several SARS-CoV-2 variants, including variants of concern (VOCs) (Table 3).

Furthermore, the performance of the M-m-qPCR was analyzed in nasopharyngeal swabs obtained from 811 individuals with respiratory infection symptoms and/or who had contact with individuals diagnosed with COVID-19. The results of the M-m-qPCR were compared with those obtained using the Molecular SARS-CoV-2 (E)—Bio-Manguinhos kit. Among the 811 participants in the survey, 502 (61.9%) were females, and 309 (38.1%) were males, with ages ranging from 5 to 85 years. Among them, 109 (13.4%) were positive for SARS-CoV-2, and 9 (1.1%) were positive for FLU-A. Notably, there was a 100% agreement between all positive and negative results obtained using the M-m-qPCR and those obtained with the commercial kit for SARS-CoV-2.

Some studies have used the melting curve strategy to detect the etiological agents of infections. Tavares et al. [52] described real-time PCR assays targeting the intergenic spacer 1 (IGS1) region from an rDNA locus to differentiate *Cryptococcus gattii sensu lato* and *Cryptococcus neoformans sensu lato*. Otaguiri et al. [31] reported a good agreement between a culture-based and a melting curve-based multiplex real-time PCR assay targeting the *cfb* gene for the detection of *Streptococcus agalactiae* in the rectal–vaginal swabs of pregnant women. Lastly, Sun et al. [53] described a high-resolution melting (HRM) multiplex assay for the direct detection of SARS-CoV-2 variants, and good agreement was observed with standard Sanger sequencing.

Our study has some limitations. (i) None of the clinical samples generated amplification signals for HRSV or HRV-B genetic markers. However, in simulated swabs saturated with synthetic mucus and spiked with the viruses, amplification signals were detected for all specific genetic markers. This result may be attributed to a reported reduction in positive cases and hospitalizations due to other respiratory viruses during the COVID-19 pandemic, as indicated in previous studies [14,15,16]. Moreover, as mentioned before, these viral agents are common causes of respiratory infections, mainly among children [23]. Thus, the lower percentage of children tested in our study may have contributed to these results. Further assays conducted during the post-COVID pandemic period will provide valuable insights into the performance of M-m-qPCR during the circulation of seasonal respiratory viruses. (ii) The LoD of the assay was determined using the synthetic plasmid (positive control), which may not fully reflect the assay sensitivity when applied to clinical samples, as the RNA purification procedure may interfere with the result. Particularly for SARS-CoV-2, assay sensitivity was similar using RNA extracted from viral cultures in Vero ATCC CCL81 cells. (iii) The probability of developing a false positive on the M-m-qPCR was not assessed with biological samples proven negative for respiratory infections. However, some precautions were taken to reduce the likelihood of false positives, including the following: (a) all primers were analyzed in silico to assess similarity with nucleotide sequences from humans and other infectious agents of respiratory infections and to identify secondary structures and dimerization potential; (b) all biological samples from the 811 individuals were processed immediately after nasopharyngeal swab collection; (c) all samples collected from the 811 individuals were simultaneously tested with the Molecular SARS-CoV-2 (E)—Bio-Manguinhos kit; (d) inconclusive tests—that is, when no amplification signal from the internal control (RNase P) was detected, the assay was repeated with a new biological sample. (iv) Only two commercial kits for detecting SARS-CoV-2 were used in our study. Therefore, we cannot generalize our findings to all real-time PCR systems. (v) The method still requires thermal cycling equipment.

Despite these limitations, our results demonstrated the following advantages: (i) the entire test, including RNA purification, sample preparation, and M-m-qPCR analysis, can be performed in about 4 h—this rapid turnaround time is crucial for ensuring timely diagnosis and patient management; (ii) the test exhibits comparable sensitivity and specificity to fluorescent probe-based NAATs; (iii) we estimated the labor costs (equipment and personal were not included) associated with nasopharyngeal swab collection and the processing of M-m-qPCR, providing insights into the economic feasibility of this method, and the value was estimated at USD 6.50; (iv) the assay can be readily adapted to a multiplex-standard PCR format, making it suitable for use in settings lacking real-time PCR equipment; and (v) as the methodology is based on a melting curve, new genetic markers for the detection of other viral agents causing respiratory infections can be incorporated into the assay.

The recent literature data report a transition from the pandemic phase of COVID-19 to an endemic state. Although mortality rates have reduced significantly, severe disease cases can occur, particularly among individuals with risk factors [54,55]. This highlights the enduring presence of SARS-CoV-2, which may co-circulate with other respiratory viral agents, and, therefore, the continuous surveillance of these viral agents remains imperative.

## 4. Patents

This study resulted in a patent application to the Brazilian National Institute of Intellectual Property (INPI-https://www.gov.br/inpi/pt-br—number BR 10 2023 017320 9) (accessed on 20 September 2023).

## Figures and Tables

**Table 1:** Characteristics of oligonucleotide primers of melting-curve-based multiplex real-time PCR.

Virus	Gene	Primers		Amplicon Size (pb)	Annealing Temperature (°C)
SARS-CoV-2	ORF1ab	RdRp-SARS-CoV-2-F	AGCTTGTCACACCGTTTCT	282	61
		RdRp-SARS-CoV-2-R	AGTCTGTGTCAACATCTCTATTTCT		
	N	NC-SARS-CoV-2-F	ACACCAATAGCAGTCCAGATG	197	61
		NC-SARS-CoV-2-R	ATTCAAGGCTCCCTCAGTTG		
FLU-A	M2	M-IAV-F	TCTAACCGAGGTCGAAACGTA	142	61
		M-IAV-R	TGGTCTTGTCTTTAGCCATTCC		
HRSV	L	RdRp-HRSV- F	AGAGAGGACCCACTAAACCA	138	61
		RdRp-HRSV-R	ATGCATACACCCAATCCAAT		
HRV-B	5′-UTR	pp-HRV-F	GTGTTCGATCAGGTGAGTTT	143	61
		pp-HRV-R	CGAGTCTTCACACCATGTC		

RNA-dependent RNA polymerase (RdRp) (ORF1ab gene) and the nucleocapsid phosphoprotein N (gene N) of SARS-CoV-2, the Matrix protein 2 (gene M2) of the Influenza A virus (FLU-A), the RdRp domain of the L protein (gene L) from Human Respiratory Syncytial Virus (HRSV), and the polyprotein (5′-UTR) from Human Rhinovirus B (HRV-B).

**Table 2:** Panel of positive controls, viruses, and microorganisms used to evaluate the melting curve-based multiplex real-time PCR specificity.

	Targets				
	RdRp-SARS-CoV-2	N-SARS-CoV-2	M2-FLU-A	L-HRSV	5′-UTR-HRV-B
Positive Control	+	+	+	+	+
SARS-CoV-2 LMM 38135	+	+	−	−	−
SARS-CoV-2 MAM 87209	+	+	−	−	−
SARS-CoV-2 HM11-20	+	+	−	−	−
SARS-CoV-2 LFL11-20	+	+	−	−	−
SARS-CoV-2 AGBCS11-20	+	+	−	−	−
SARS-CoV-2 PQB12-20	+	+	−	−	−
SARS-CoV-2 AOC12-20	+	+	−	−	−
SARS-CoV-2 CNPB11-20	+	+	−	−	−
SARS-CoV-2 MPC12-20	+	+	−	−	−
SARS-CoV-2 MKA01-21	+	+	−	−	−
SARS-CoV-2 BWS02-21	+	+	−	−	−
SARS-CoV-2 VCS02-21	+	+	−	−	−
SARS-CoV-2 EOC02-21	+	+	−	−	−
SARS-CoV-2 SMGC02-21	+	+	−	−	−
SARS-CoV-2 VRS02-21	+	+	−	−	−
SARS-CoV-2 SS09-21	+	+	−	−	−
SARS-CoV-2 ARM09-21	+	+	−	−	−
SARS-CoV-2 CHB09-21	+	+	−	−	−
SARS-CoV-2 ALMO12-21	+	+	−	−	−
SARS-CoV-2 MPV10-21	+	+	−	−	−
SARS-CoV-2 DIBM12-21	+	+	−	−	−
SARS-CoV-2 PLCSC01-22	+	+	−	−	−
Human Rhinovirus HRV 3760	−	−	−	−	+
Human Rhinovirus HRV HRV01	−	−	−	−	+
Human Rhinovirus HRV HRV02	−	−	−	−	+
Human Respiratory Syncytial Virus HRSV 3760	−	−	−	+	−
Human Respiratory Syncytial Virus HRSV 4226	−	−	−	+	−
Human Respiratory Syncytial Virus HRSV 4122	−	−	−	+	−
Human Respiratory Syncytial Virus HRSV IVC1	−	−	−	+	−
Human Respiratory Syncytial Virus HRSV IVC2	−	−	−	+	−
Human Respiratory Syncytial Virus HRSV IVC3	−	−	−	+	−
Influenza Virus A FLU-A/H1N1 FLU	−	−	+	−	−
Influenza Virus A FLU-A/H3N2	−	−	+	−	−
Influenza Virus A FLU-A CS1	−	−	+	−	−
Influenza Virus A FLU-A CS2	−	−	+	−	−
Influenza Virus A FLU-A CS3	−	−	+	−	−
Influenza Virus A FLU-A CS4	−	−	+	−	−
Influenza Virus A FLU-A CS5	−	−	+	−	−
Influenza Virus B	−	−	−	−	−
Human Enterovirus (HEV)	−	−	−	−	−
Seasonal Coronavirus (HCoVs)	−	−	−	−	−
Parainfluenza Virus 1 (HPIV1)	−	−	−	−	−
Parainfluenza Virus 2 (HPIV2)	−	−	−	−	−
Parainfluenza Virus 3 (HPIV3)	−	−	−	−	−
Parainfluenza Virus 4 (HPIV4)	−	−	−	−	−
Human Adenovirus ADV 3226	−	−	−	−	−
Human Adenovirus ADH 4122	−	−	−	−	−
*Staphylococcus aureus* ATCC 25923	−	−	−	−	−
*Staphylococcus epidermidis* ATCC 35984	−	−	−	−	−
*Staphylococcus haemolyticus* ATCC 29968	−	−	−	−	−
*Staphylococcus saprophyticus* ATCC 15305	−	−	−	−	−
*Staphylococcus pseudintermedius* SIG34	−	−	−	−	−
*Staphylococcus schleiferi*	−	−	−	−	−
*Pseudomonas aeruginosa* ATCC 27858	−	−	−	−	−
*Klebsiella pneumonia* ATCC 10031	−	−	−	−	−
*Klebsiella pneumoniae* kp39	−	−	−	−	−
*Enterococcus faecalis* ATCC 51299	−	−	−	−	−
*Enterococcus faecium* ATCC 6569	−	−	−	−	−
*Escherichia coli* ATCC 25922	−	−	−	−	−
*Candida albicans* ATCC 25923	−	−	−	−	−
*Candida glabrata* ATCC 2001	−	−	−	−	−
*Candida krusei* ATCC 34135	−	−	−	−	−
*Candida parapsilosis* ATCC 22019	−	−	−	−	−
*Candida tropicalis* ATCC 28707	−	−	−	−	−
*Candida auris* 10913	−	−	−	−	−
*Cryptococcus neoformans* ATCC 34872	−	−	−	−	−
*Cryptococcus gattii* ATCC 32269	−	−	−	−	−
*Paracoccidioides brasiliensis* Pb1 8	−	−	−	−	−
*Histoplasma capsulatum* I	−	−	−	−	−

**Table 3:** Panel of different variants of SARS-CoV-2 used to validate the performance of the melting-curve-based multiplex real-time PCR specificity.

Date	Viral Strain	Pango Lineage
2020-11	SARS-CoV-2 LAVIR-HM-UEL	B.1.1.33
2020-11	SARS-CoV-2 LAVIR-AB-UEL	B.1.1.143
2020-11	SARS-CoV-2 LAVIR-PB-UEL	B.1.1.28
2020-11	SARS-CoV-2 LAVIR-LL-UEL	B.1.1.33
2020-11	SARS-CoV-2 LAVIR-CB-UEL	P.2
2020-12	SARS-CoV-2 LAVIR-AC-UEL	B.1.1.28
2020-12	SARS-CoV-2 LAVIR-MC-UEL	P.2
2021-01	SARS-CoV-2 LAVIR-MA-UEL	P.2
2021-02	SARS-CoV-2 LAVIR-BS-UEL	B.1.1.7
2021-02	SARS-CoV-2 LAVIR-VCS-UEL	B.1.1.7
2021-02	SARS-CoV-2 LAVIR-SC-UEL	B.1.1.7
2021-02	SARS-CoV-2 LAVIR-VRS-UEL	B.1.1.7
2021-02	SARS-CoV-2 LAVIR-EC-UEL	B.1.1.7
2021-09	SS09-21	Delta (B.1.617.2-like)
2021-09	ARM09-21	Delta (B.1.617.2-like)
2021-09	CHB09-21	Delta (B.1.617.2-like)
2021-12	ALMO12-21	Omicron (BA.1-like)
2021-10	MPV10-21	Delta (B.1.617.2-like)
2021-12	DIBM12-21	Omicron (BA.1-like)
2022-01	PLCSC01-22	Delta (B.1.617.2-like)

Date: Clinical sample collection period. Viral strain: Nasopharyngeal specimens collected from COVID-19-positive patients (CT < 21) diagnosed with TaqPath™ COVID-19 CE-IVD RT-PCR kit. Pango lineage: Defined according to the Spike protein-encoding gene sequence analysis.

## Data Availability

Data are contained within the article and Supplementary Materials.

## Supplementary Materials

The following supporting information can be downloaded at https://www.mdpi.com/article/10.3390/microorganisms11112692/s1: Figure S1: Construction of pUC57 (positive controls) carrying the consensus sequences of target genes used in melting-curve-based multiplex real-time PCR. (**a**) RNA-dependent RNA polymerase (RdRp) and the nucleocapsid phosphoprotein N of SARS-CoV-2; (**b**) Matrix protein 2 of Influenza A virus (FLU-A); (**c**) and the RdRp domain of the L protein from Human Respiratory Syncytial Virus (HRSV) and the 5′-UTR polyprotein from Human Rhinovirus B (HRV-B). Figure S2: Monoplex real-time PCR for determining melting temperature peaks (Tms) of RNA-dependent RNA polymerase (RdRp) and the nucleocapsid phosphoprotein (N) of SARS-CoV-2, Matrix protein 2 (M2) of the Influenza A virus (FLU-A), the RdRp domain of the L protein (L) from Human Respiratory Syncytial Virus (HRSV), and the 5′-UTR polyprotein from Human Rhinovirus B (HRV-B) amplicons using positive (plasmid) controls. RNase P amplicon Tm peak was determined using human nasopharyngeal swab samples. Figure S3: Performance of real-time PCR assay for SARS-CoV-2. Melting temperature peaks (Tms) of (**a**) RNA-dependent RNA polymerase (RdRp) and (**c**) nucleocapsid phosphoprotein N of SARS-CoV-2 (**c**) using SARS-CoV-2/human/BRA/SP02cc/2020 culture in Vero ATCC CCL81 cells. Sensitivity of real-time PCR assays: Amplification pattern of serial dilution corresponding to 10,000, 1000, 100, 10, and 5 viral particles. The virus titration was determined using a tissue culture infectious dose (TCID_50_/mL) assay [37]. (**b**) RNA-dependent RNA polymerase (RdRp) and (**d**) nucleocapsid phosphoprotein N.
