# Supplementary material for: Development of a Melting-Curve-Based Multiplex Real-Time PCR Assay for the Simultaneous Detection of Viruses Causing Respiratory Infection
Source: Microorganisms. 2023 Nov 2;11(11):2692. doi: 10.3390/microorganisms11112692 (PMC10672821; doi:10.3390/microorganisms11112692)
Supplement: Supplementary file 1 [file microorganisms-11-02692-s001.zip › microorganisms-2657792-supplementary.pdf]

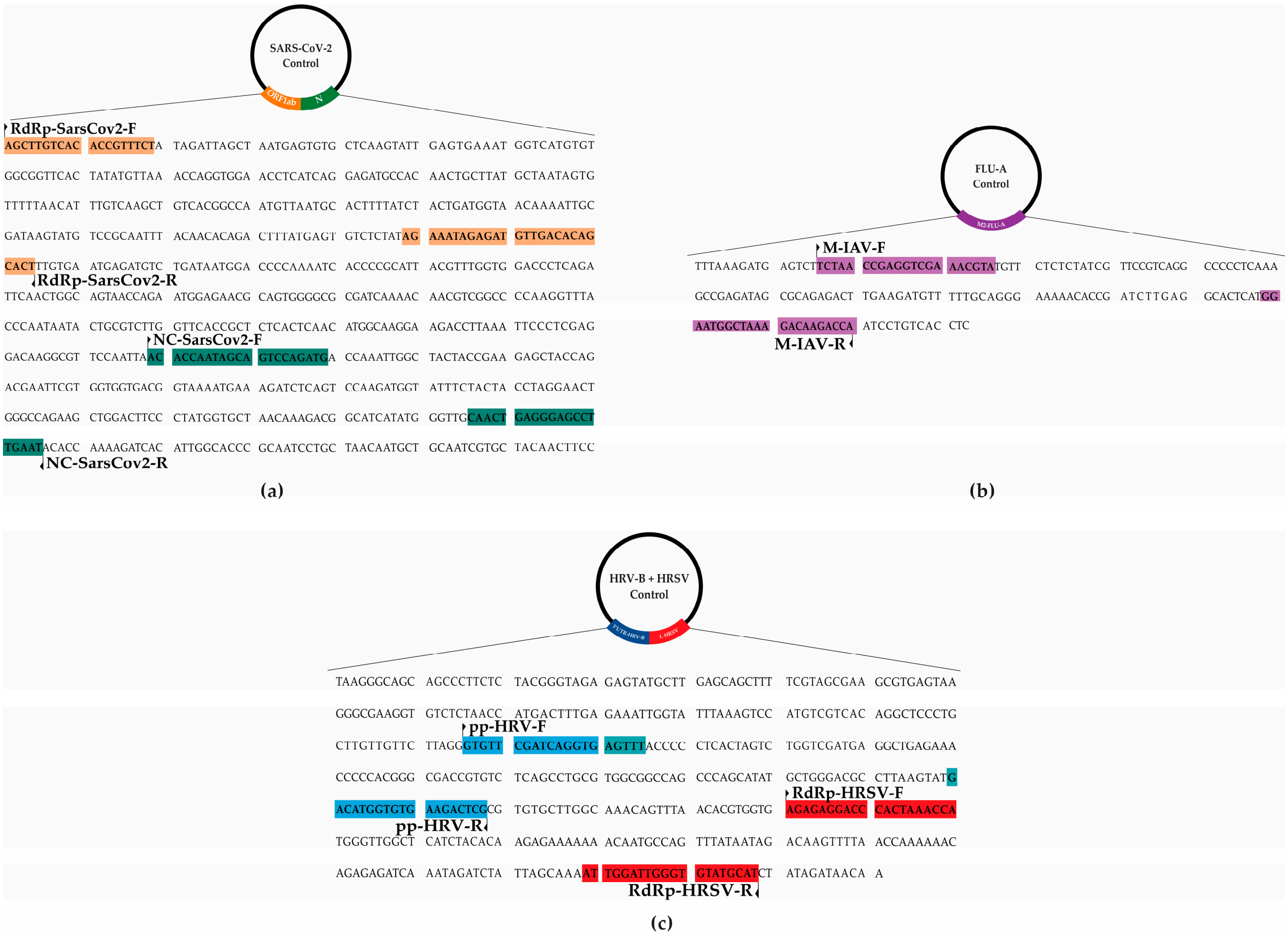


**Figure S1.** Construction of pUC57 (positive controls) carrying the consensus sequences of target genes used in melting curve-based multiplex real-time PCR. **(a)** RNA-dependent RNA polymerase (RdRp) and the nucleocapsid phosphoprotein N of SARS-CoV-2; **(b)** Matrix protein 2 of Influenza A virus (FLU-A); **(c)** and the RdRp domain of L protein from Human Respiratory Syncytial Virus (HRSV), and the 5′-UTR polyprotein from Human Rhinovirus B (HRV-B).

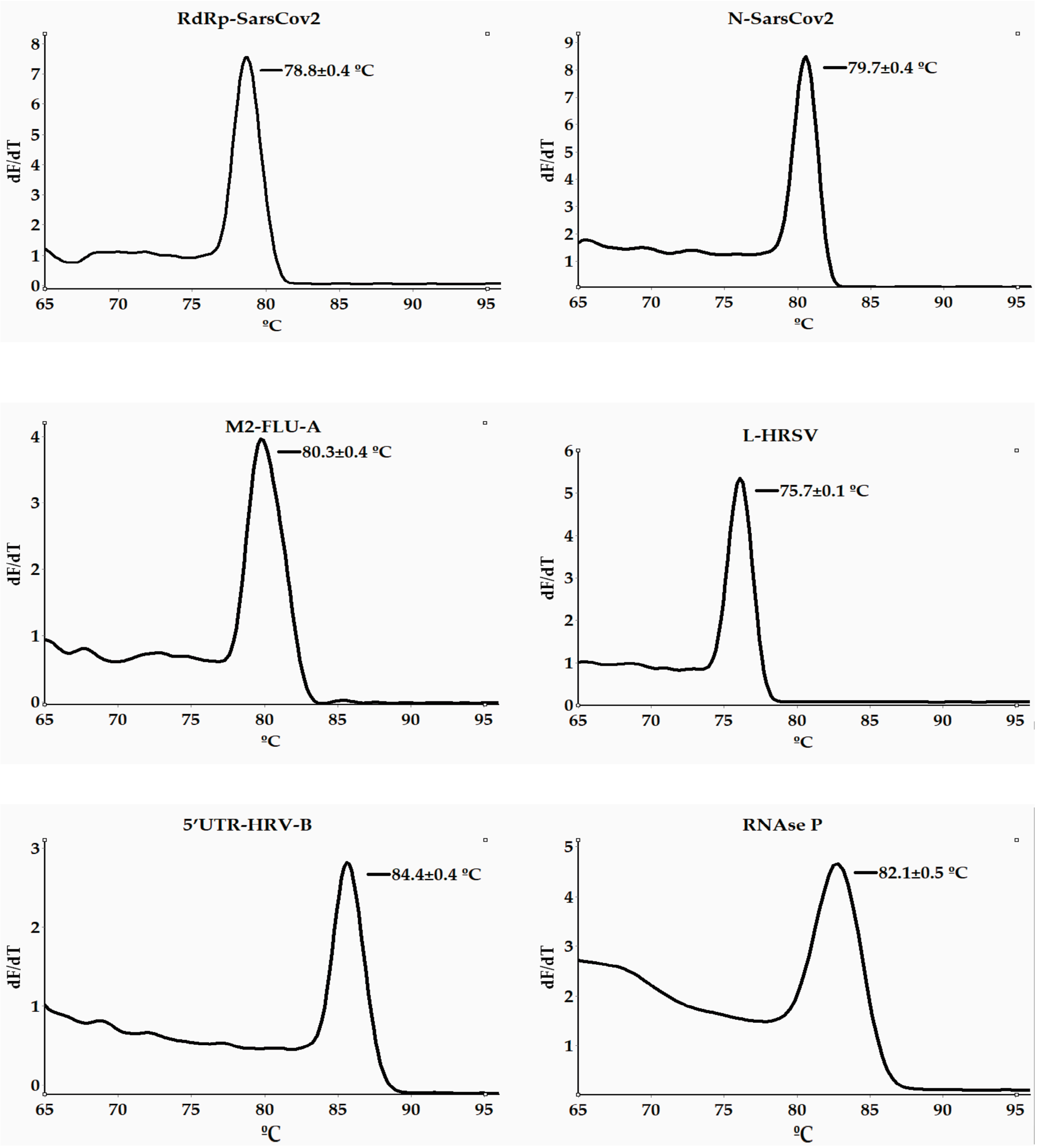


**Figure S2.** Monoplex real-time PCR for determining melting temperature peaks (Tm) of RNA-dependent RNA polymerase (RdRp) and the nucleocapsid phosphoprotein (N) of SARS-CoV-2, the Matrix protein 2 (M2) of Influenza A virus (FLU-A), the RdRp domain of L protein (L) from Human Respiratory Syncytial Virus (HRSV), the 5′-UTR polyprotein from Human Rhinovirus B (HRV-B) amplicons using positive (plasmid) controls. RNAse P amplicon Tm peak was determined using human nasopharyngeal swab samples.

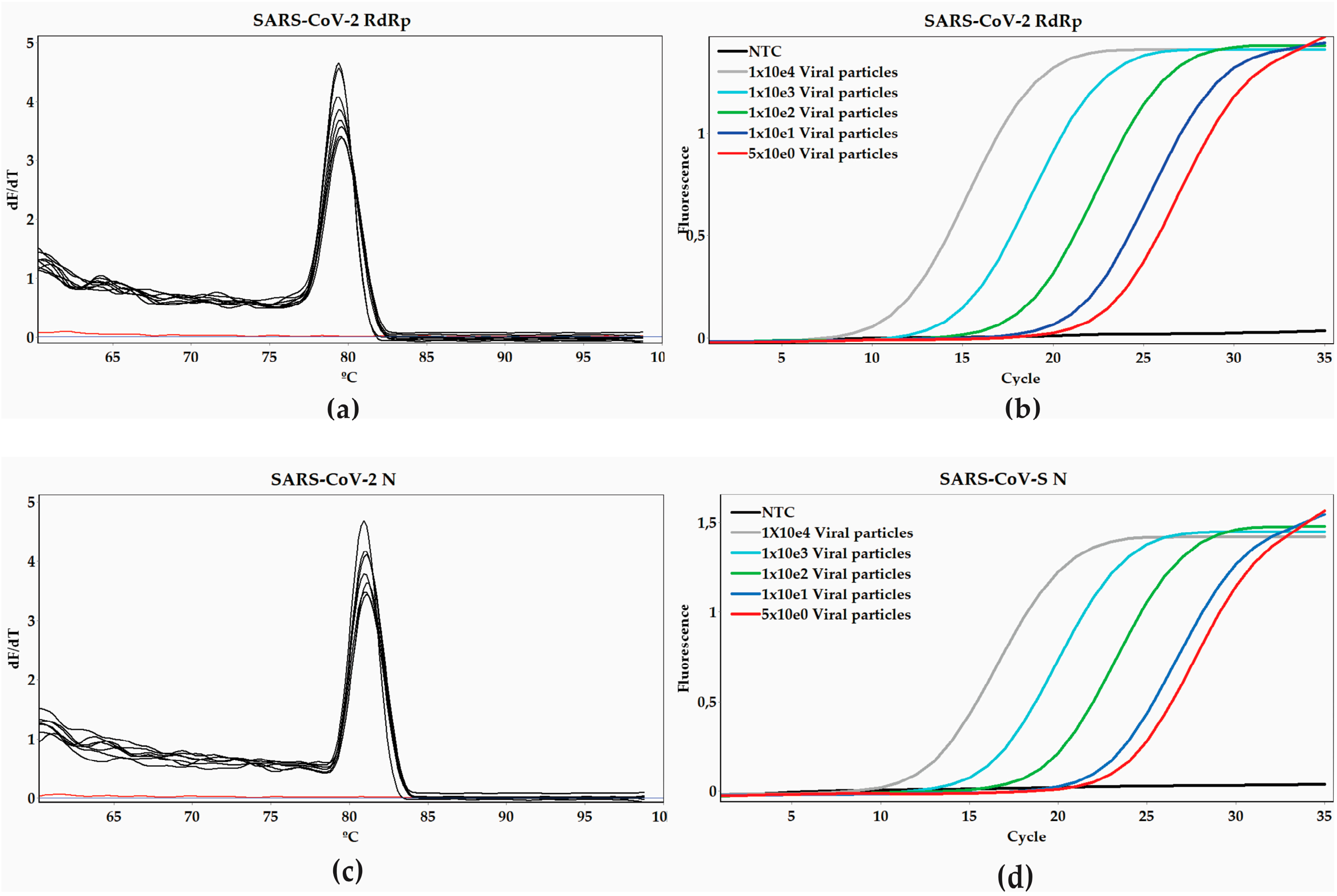


**Figure S3.** Performance of real-time PCR assay for SARS-CoV-2. Melting temperature peaks (Tm) of (**a**) RNA-dependent RNA polymerase (RdRp) and (**c**) nucleocapsid phosphoprotein N of SARS-CoV-2 **(c)** using SARS-CoV-2/human/BRA/SP02cc/2020 culture in Vero ATCC CCL81 cells. Sensitivity of real-time PCR assays: Amplification pattern of serial dilution corresponding to 10000, 1000, 100, 10, and 5 viral particles. The virus titration was determined by tissue culture infectious dose ($TCID_{50}$/mL) assay [37]. (**b**) RNA-dependent RNA polymerase (RdRp) and (**d**) nucleocapsid phosphoprotein N.
